# Anti-ADAMTS13 Antibodies and a Novel Heterozygous p.R1177Q Mutation in a Case of Pregnancy-Onset Immune-Mediated Thrombotic Thrombocytopenic Purpura

**DOI:** 10.1055/s-0037-1615252

**Published:** 2018-01-08

**Authors:** Elien Roose, Claudia Tersteeg, Ruth Demeersseman, An-Sofie Schelpe, Louis Deforche, Inge Pareyn, Aline Vandenbulcke, Nele Vandeputte, Daan Dierickx, Jan Voorberg, Hans Deckmyn, Simon F. De Meyer, Karen Vanhoorelbeke

**Affiliations:** 1 Laboratory for Thrombosis Research, IRF Life Sciences, KU Leuven Campus Kulak Kortrijk, Kortrijk, Belgium; 2 Department of Hematology, University Hospitals Leuven, Leuven, Belgium; 3 Department of Plasma Proteins, Sanquin-Academic Medical Center Landsteiner Laboratory, Amsterdam, The Netherlands

**Keywords:** thrombotic thrombocytopenic purpura, pregnancy-onset TTP, SNPs and mutations, anti-ADAMTS13 autoantibodies

## Abstract

In this study, we investigated a case of pregnancy-onset thrombotic thrombocytopenic purpura (TTP). The patient had severely decreased ADAMTS13 ( *a* *d* isintegrin *a* nd *m* etalloprotease with *t* hrombo *s* pondin type 1 motif, member 13) activity levels during acute phase and the presence of inhibitory anti-ADAMTS13 autoantibodies was demonstrated, which led to the diagnosis of immune-mediated TTP. However, ADAMTS13 activity was only mildly restored during remission, although inhibitory anti-ADAMTS13 antibodies were no longer detected. We hypothesized that genetic abnormalities could account for this discrepancy between ADAMTS13 activity and antigen. Genetic analysis revealed the presence of two heterozygous substitutions on the same allele: a single nucleotide polymorphism (SNP) c.2699C > T (p.A900V), located in the beginning of the T5 domain, and a mutation c.3530G > A (p.R1177Q) located in the third linker region of ADAMTS13. In vitro testing of those substitutions by expression of recombinant proteins revealed a normal secretion but a reduced ADAMTS13 activity by the novel p.R1177Q mutation, which could partially explain the subnormal activity levels found during remission. Although changes in the linker region might induce conformational changes in ADAMTS13, the p.R1177Q mutation in the third linker region of ADAMTS13 did not expose a cryptic epitope in the metalloprotease domain. In conclusion, we report on an immune-mediated pregnancy-onset TTP patient who had inhibitory anti-ADAMTS13 autoantibodies during acute phase, but not during remission. Genetic analysis confirmed the diagnosis of immune-mediated TTP and revealed the novel p.R1177Q mutation which mildly impaired ADAMTS13 activity.

## Introduction

The rare and life-threatening disease thrombotic thrombocytopenic purpura (TTP) is caused by a severe deficiency in the metalloprotease ADAMTS13 ( *a* *d* isintegrin *a* nd *m* etalloprotease with *t* hrombo *s* pondin type 1 motif, member 13). 1 ADAMTS13 is encoded by 29 exons which results in a multidomain protein containing a signal peptide, propeptide, metalloprotease domain, disintegrin-like domain, a first thrombospondin type 1 repeat (T1), cysteine-rich domain, spacer domain, seven additional T domains (T2-T8), and two CUB domains. 2

Inefficient functioning of ADAMTS13 (< 10% activity) results in the accumulation of ultra-large von Willebrand factor (UL-VWF) multimers in circulation. UL-VWF is hyper reactive and spontaneously binds platelets. This leads to the formation of microthrombi that obstruct the microvasculature, resulting in organ failure, thrombocytopenia, and hemolytic anemia or even death when left untreated. 1 In more than 95% of the patients, TTP is caused by the presence of anti-ADAMTS13 autoantibodies (immune-mediated TTP) that inhibit ADAMTS13 activity or accelerate its clearance from circulation. 3 4 Far less patients (< 5%) suffer from congenital TTP (Upshaw-Schulman syndrome), in which ADAMTS13 deficiency is due to genetic mutations within the *ADAMTS13* gene. 5 Those mutations can lead to a secretion deficiency or impaired activity of the enzyme. 6 The onset of TTP is not simply caused by an ADAMTS13 deficiency alone, but an additional trigger like infection or pregnancy is typically needed to initiate an acute TTP episode.

The diagnosis of immune-mediated TTP at presentation is often based on the presence of anti-ADAMTS13 autoantibodies and the absence of a family history, whereas the opposite holds true for congenital TTP. However, some rare cases of congenital TTP patients presenting with anti-ADAMTS13 autoantibodies have been described. 7 8 9 On the other hand, two sisters have been reported who had acquired immune-mediated TTP, although their familial history was suspicious for congenital TTP. 10 Hence, it was suggested that investigating the presence of both anti-ADAMTS13 autoantibodies and *ADAMTS13* genetic variations is sometimes essential to improve the differential diagnosis between immune-mediated TTP and congenital TTP. 11 In addition, some patients with immune-mediated TTP have been described with a heterozygous mutation (R1060W) in their *ADAMTS13* gene. 9 In vitro studies of the heterozygous mutations found in immune-mediated TTP patients might help in understanding ADAMTS13 activity levels during remission or might aid in further unravelling the mode of action of ADAMTS13.

In this study, we investigated a case of pregnancy-onset TTP that presented with anti-ADAMTS13 autoantibodies and also had a heterozygous known ADAMTS13 SNP (p.A900V, exon 21) together with a novel mutation (p.R1177Q, exon 25). The influence on the secretion, activity, and conformation of ADAMTS13 was investigated.

## Materials and Methods

### Case Report

A 27-year-old woman was admitted to the hospital (November 2012) in the 20th week of her pregnancy where she was diagnosed with an acute pregnancy-onset TTP episode (one acute phase sample available before treatment [Ac]). Clinical symptoms were arterial hypertension and the presence of petechiae, but no neurological symptoms. Standard laboratory analysis revealed thrombocytopenia (5 × 10 ^9^ platelets/L), hemolytic anemia (hemoglobin 7.0 g/dL) with presence of schistocytes (7–10/1,000 red blood cells), and increased lactate dehydrogenase levels (454 U/L). The direct Coombs test was negative. Due to severe intrauterine growth restriction of the fetus, her pregnancy was terminated. Therapeutic plasma exchange (PEx) was given in combination with corticosteroids and platelet count normalized after 6 days. MRI of the brain showed old ischemic lesions in both cerebral hemispheres, probably from an ischemic event 7 months earlier. At that time, she also had a missed abortion, making pregnancy termination necessary. PEx was restarted for 7 consecutive days after an interval of 2 days because of recurrent thrombocytopenia. In December 2012, PEx was reinitiated again, but was rapidly stopped following diagnosis of *Escherichia coli* –induced sepsis. From that moment, she has been in remission (two remission samples available: February 2013, sample remission 1 [R1] and October 2014, sample remission 2 [R2]). ADAMTS13 was fully characterized both in plasma and on a molecular level to determine the cause of this pregnancy-onset TTP episode (see results). The patient gave informed consent according to the Declaration of Helsinki. The parents and other family members were unavailable for the study.

### ADAMTS13 Activity

ADAMTS13 activity levels of the patient plasma samples and recombinant human ADAMTS13 proteins (rhADAMTS13) were measured using the FRETS-VWF73 substrate (Peptides International, Louisville, Kentucky, United States) with normal human plasma pool (NHP; 0.01, 0.02, and 0.03 µg/mL) as a standard curve as previously described. 12 13 All ADAMTS13 activities were measured in three separate experiments, and in each experiment five different ADAMTS13 concentrations were used.

### ADAMTS13 Antigen

Plasma ADAMTS13 or rhADAMTS13 antigen levels were determined using an in-house developed ADAMTS13 antigen ELISA, as described before with minor modifications. 14 15 Briefly, the in-house developed anti-ADAMTS13 antibody 3H9 (5 µg/mL) was coated and patient plasma (15 V%), non-diluted expression medium containing rhADAMTS13, purified rhADAMTS13, or its variants (1/250, 1/500 and 1/800 dilutions) were added in a 1.5 over 2.5 dilution series. Captured ADAMTS13 was detected using two in-house developed biotinylated anti-ADAMTS13 antibodies 17G2 and 19H4 (1 µg/mL each), followed by HRP-labeled streptavidin (Roche Diagnostics, Mannheim, Germany). The colorimetric reaction was initiated by addition of o-phenylenediamine (OPD) and H _2_ O _2_ , stopped with 4M sulfuric acid, and the absorbance was measured at 490 nm. NHP was used as a reference and set as 1 µg/mL.

### Identification of Inhibitory Anti-ADAMTS13 Autoantibodies

An in-house developed anti-ADAMTS13 antibody ELISA was performed to detect anti-ADAMTS13 IgG antibodies present in the patient plasma samples. First, rhADAMTS13 (15 nM) in phosphate buffered saline (PBS) was coated onto a 96-well microtiter plate. After blocking (3% milk in PBS), the patient plasma samples (diluted 1/20) were added in a half serial dilution (0.3% milk in PBS, 1 hour, 37°C). Anti-ADAMTS13 antibodies were detected using a mixture of HRP-labeled antihuman IgG _1_ (1/20,000; Sanquin Division Reagents, Amsterdam, the Netherlands), antihuman IgG _2_ , IgG _3_ , and IgG _4_ antibodies (1/2,000; Sanquin Division Reagents). The colorimetric reaction was performed as described earlier.

Presence of anti-ADAMTS13 autoantibodies was also analyzed through sodium dodecyl sulfate polyacrylamide gel electrophoresis (SDS-PAGE) and Western blot as previously described with minor modifications. 16 Briefly, rhADAMTS13 (2 µg) was loaded and a 7.5% SDS-PAGE was performed. Samples were transferred to a Roti-polyvinylidene fluoride membrane (Carl Roth GmbH, Karlsruhe, Germany). After blocking with 5% milk in Tris buffered saline (TBS), the membrane was incubated with different plasma samples (diluted 1/100) in 5% milk in TBS and 0.05% Tween-20. The previously described recombinant human anti-ADAMTS13 antibody II-1 was used as a positive control. 17 18 Bound antibodies were detected with a mixture of HRP-labeled goat antihuman IgA (Abcam, Cambridge, UK; diluted 1/10,000 in 0.3% milk in TBS) and rabbit antihuman IgG and IgM antibodies (Jackson ImmunoResearch, Suffolk, UK; diluted 1/10,000 in 0.3% milk in TBS). Finally, the membrane was incubated for 5 minutes with 2 mL of the SuperSignal West Pico Chemiluminescent Substrate kit (Acros Organics, Geel, Belgium) and bands were visualized with a Fujifilm LAS-4000 device (Fujifilm, Tokyo, Japan).

The presence of inhibitory anti-ADAMTS13 autoantibodies was determined using a mixing experiment as described previously. 19 20 Briefly, patient plasma samples were heat inactivated (1 hour, 56°C) to inhibit residual ADAMTS13 activity, and next incubated with NHP (containing active ADAMTS13, 50 V%) for 2 hours at room temperature. Finally, the mixed plasma samples were analyzed for their residual ADAMTS13 activity using the FRETS-VWF73 substrate. 19 20

### Identification of Substitutions in *ADAMTS13*

Genomic DNA was extracted from peripheral leukocytes using the salting out procedure as described. 21 All 29 exons and exon–intron boundaries of *ADAMTS13* were amplified by polymerase chain reaction (PCR) as described before with primers as described elsewhere, 13 22 23 and the sequence of each product was determined (GATC Biotech AG, Konstanz, Germany).

To determine whether polymorphisms and mutations are located on the same allele, exons 21 till 25 of *ADAMTS13* were amplified with the help of the Elongase Enzyme mix (Invitrogen, Carlsbad, California, United States). Next, fragments were ligated in TOPO vectors according to the TOPO XL PCR cloning kit (Invitrogen). After transformation, plasmid DNA was extracted with the Qiaprep Spin Miniprep Kit (Qiagen, Venlo, the Netherlands) and sent for sequencing.

### Expression of Wild Type (WT), p.A900V, p.R1177Q, and p.A900V/R1177Q ADAMTS13

The QuickChange XL Site-Directed Mutagenesis kit (Stratagene, La Jolla, California, United States) was used to introduce the identified substitutions c.2699C > T (p.A900V), c.3530G > A (p.R1177Q), and c.2699C > T/c.3530G > A (p.A900V/p.R1177Q) in the pcDNA6.1-ADAMTS13-WT expression vector containing a C-terminal V5 and His-tag. 13 After sequencing the constructs (GATC Biotech AG), the Qiagen Plasmid Mega Kit (Qiagen) was used to produce sufficient amounts of the four different plasmids (pcDNA6.1-ADAMTS13-WT, pcDNA6.1-ADAMTS13-p.A900V, pcDNA6.1-ADAMTS13-p.R1177Q, and pcDNA6.1-ADAMTS13-p.A900V/p.R1177Q).

The effect of the mutations on ADAMTS13 expression levels was studied by transient transfection of the four different plasmids in Chinese Hamster Ovary (CHO) cells together with a GFP expression plasmid (pmaxGFP, Lonza, San Diego, California, United States) to correct for transfection efficiency. 13 24 Plasmids were transfected under homozygous (pcDNA6.1-ADAMTS13-WT, pcDNA6.1-ADAMTS13-p.A900V, pcDNA6.1-ADAMTS13-p.R1177Q, pcDNA6.1-ADAMTS13-p.A900V/p.R1177Q) or heterozygous (pcDNA6.1-ADAMTS13-p.A900V + pcDNA6.1-ADAMTS13-p.R1177Q) conditions. Briefly, CHO cells were grown in Kaighn's modification of Ham's F-12 medium (F-12K, ATCC, Molsheim, France) with 10% FCS and 1% Antibiotic-Antimycotic (Invitrogen) until 60 to 80% confluency. JetPRIME Polyplus transfection reagent (VWR International, Radnor, Pennsylvania, United States) was used to transfect cells with a total of 4 µg plasmid DNA (homozygous pcDNA6.1-ADAMTS13-WT/pmaxGFP, pcDNA6.1-ADAMTS13-p.A900V/ pmaxGFP, pcDNA6.1-ADAMTS13-p.R1177Q/ pmaxGFP, and pcDNA6.1-ADAMTS13-p.A900V/p.R1177Q/ pmaxGFP: 3.6 µg/0.4 µg or heterozygous pcDNA6.1-ADAMTS13-p.A900V/pcDNA6.1-ADAMTS13-p.R1177Q/pmaxGFP: 1.8 µg/1.8 µg/0.4 µg). To determine transfection efficiency, GFP expressing cells were visualized with a Nikon eclipse TE200 inverted fluorescence microscope (Nikon Instruments, Melville, New York, United States) after 24 hours. Three pictures of each condition were taken, and transfection efficiency was defined as the percentage of fluorescent cells present. Next, expression medium was harvested to determine the ADAMTS13 expression levels, as described above.

Stable cell lines were also generated to produce sufficient amounts of ADAMTS13-WT, ADAMTS13-p.A900V, ADAMTS13-p.R1177Q, and ADAMTS13-p.A900V/p.R1177Q. Human embryonic kidney (HEK) 293T cells were stably transfected with the four different plasmid DNA constructs (pcDNA6.1-ADAMTS13-WT, pcDNA6.1-ADAMTS13-p.A900V, pcDNA6.1-ADAMTS13-p.R1177Q, and pcDNA6.1-ADAMTS13-p.A900V/p.R1177Q) using JetPRIME Polyplus transfection reagent. Cells were selected on Blasticidin (Invitrogen). The stable cells were cultured for 3 days after which the medium was harvested and centrifuged to remove cell debris (20 minutes, 10,000 rpm, 4°C).

### Purification of rhADAMTS13 and Its Variants

rhADAMTS13 and its variants were purified using Zn ^2+^ affinity chromatography on an ÄKTA device (GE Healthcare, Waukesha, Wisconsin, United States). First, expression medium was added to a 4MA ultrafiltration hollow fiber cartridge (GE, Healthcare) to concentrate and dialyze the conditioned medium against the appropriate binding buffer (20 mM imidazole, 20 mM NaH _2_ PO _4_ .H _2_ O, 500 mM NaCl, pH 7.4). After loading the sample on the Zn ^2+^ coupled HisTrap HP column (GE Healthcare), an imidazole containing buffer (500 mM imidazole, 20 mM NaH _2_ PO _4_ .H _2_ O, 500 mM NaCl, pH 7.4) was used for elution. Positive fractions were pooled and dialyzed against HEPES-buffered saline (50 mM HEPES, 5 mM CaCl _2_ .2H _2_ O, 1 μM ZnCl _2_ , 150 mM NaCl). Concentrations were determined in the ADAMTS13 antigen ELISA as described above.

### Exposure of a Cryptic Epitope in rhADAMTS13

To study whether the polymorphism and the mutation resulted in exposure of a cryptic epitope in ADAMTS13, 96-well microtiter plates were coated with the monoclonal mouse anti-human ADAMTS13 antibody 6A6 (5 µg/mL), which recognizes a cryptic epitope in the metalloprotease domain of ADAMTS13. 25 Recombinant proteins (100 nM; WT, p.A900V, p.R1177Q and p.A900V/p.R1177Q) were added in a 1 over 2 dilution series. Bound protein was detected with HRP-labeled anti-V5 antibodies. The colorimetric reaction was performed as described above. MDTCS (ADAMTS13 without T2 till CUB domains, and with the cryptic epitope in the metalloprotease domain exposed 25 ) was used as a positive control.

### Statistical Analysis

All data are presented as mean ± standard deviation. GraphPad Prism v5.03 software (GraphPad Software, San Diego, California, United States) was used for statistical analysis through Kruskal–Wallis tests for comparison of WT with the other groups. A *p* -value of <0.05 was considered significant.

## Results

### ADAMTS13-Related Parameters in Acute and Remission Samples

**Fig. 1:**
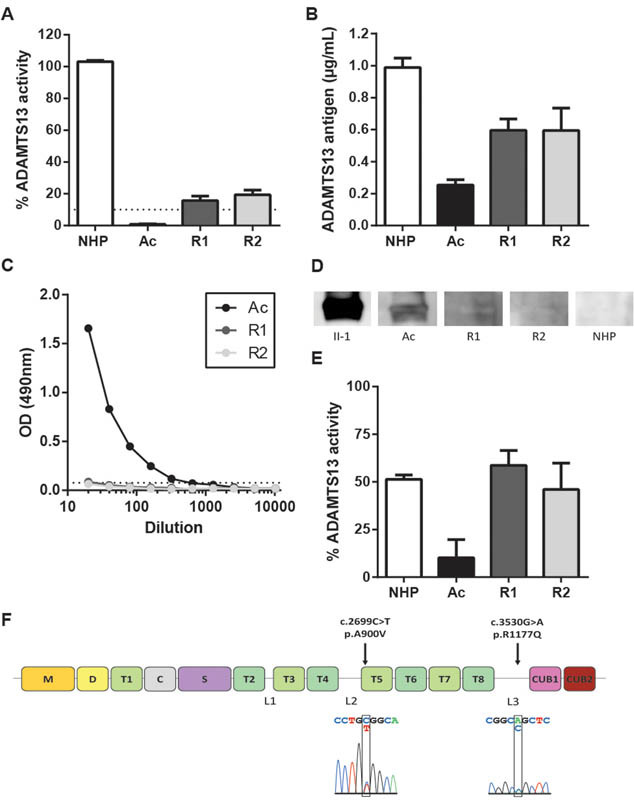
ADAMTS13 activity, antigen, anti-ADAMTS13 autoantibodies and gene analysis from the TTP patient. Patient plasma samples of acute (Ac) and remission phases (one plasma sample just after the acute episode [remission 1, R1] and one plasma sample almost 2 years after achieving complete remission [remission 2, R2]) were analyzed. ( **A** ) ADAMTS13 activity was measured using the FRETS-VWF73 assay. NHP was used as a reference and set as 100% ADAMTS13 activity. Below 10% (dotted line) indicates severely decreased activity ( *n* = 3). ( **B** ) Plasma ADAMTS13 antigen levels were measured using ELISA. NHP was used as a reference and set as 1 µg/mL ( *n* = 3). ( **C** ) Anti-ADAMTS13 autoantibodies were detected by adding plasma to wells coated with rhADAMTS13. The dotted line indicates the background ( *n* = 1). ( **D** ) Western blot was also used to detect anti-ADAMTS13 autoantibodies. rhADAMTS13 was loaded onto an SDS-polyacrylamide gel. After transfer, the membrane containing rhADAMTS13 was incubated with plasma samples, or purified human anti-ADAMTS13 antibody II-1 as a positive control. ( **E** ) A mixing experiment was performed to detect inhibitory anti-ADAMTS13 autoantibodies in plasma from the patient. Heat-inactivated patient plasma was added to fresh NHP (50 V%) and residual ADAMTS13 activity was determined using the FRETS-VWF73 assay ( *n* = 3). ( **F** ) Representation of the domain structure of ADAMTS13 with the metalloprotease domain (M), disintegrin-like domain (D), a first thrombospondin type 1 repeat (T1), cysteine-rich domain (C), spacer domain (S), seven additional T domains (T2-T8), and 2 CUB domains. The three flexible linker regions are indicated by L1, L2, and L3. The SNP c.2699C > T (p.A900V) is located in the beginning of the T5 domain, while the mutation c.3530G > A (p.R1177Q) is located in the third linker region between T8 and CUB1 domains. The sequence chromatograms are shown below the ADAMTS13 domain structure and show the DNA sequence of the SNP and the mutation and their heterozygous nature.

First, we wanted to unequivocally assess whether the pregnancy-onset TTP was related to an immune-mediated or a congenital deficiency in ADAMTS13. We therefore determined ADAMTS13 activity and antigen levels, presence of anti-ADAMTS13 antibodies, and presence of mutations and/or SNPs in the *ADAMTS13* gene. During the acute phase, ADAMTS13 activity levels were severely decreased (0.78 ± 0.30%; Fig. 1A , Ac), which was in line with the detection of low ADAMTS13 antigen levels (0.27 ± 0.06 µg/mL; Fig. 1B , Ac). Anti-ADAMTS13 autoantibodies were detected in the plasma of the patient during the acute phase, using both ELISA and Western blot ( Fig. 1C, D , Ac) leading to an initial diagnosis of immune-mediated TTP. In addition, the mixing experiment showed that the patients' plasma contained inhibitory anti-ADAMTS13 autoantibodies, since ADAMTS13 activity from NHP could be inhibited by addition of heat inactivated acute phase plasma from the patient ( Fig. 1E , Ac). As expected, during remission, ADAMTS13 activity ( Fig. 1A , R1 and R2) and antigen ( Fig. 1B , R1 and R2) increased and anti-ADAMTS13 autoantibodies disappeared ( Fig. 1C, D , R1 and R2).

The limited increase in ADAMTS13 activity (15.64 ± 2.92% and 19.33 ± 3.05% for R1 and R2, respectively; Fig. 1A ) as compared with the larger increase in ADAMTS13 antigen levels (R1: 0.58 ± 0.06 µg/mL; R2: 0.59 ± 0.13 µg/mL; Fig. 1B ), could not be explained by the presence of detectable inhibitory anti-ADAMTS13 autoantibodies during remission ( Fig. 1E ). Indeed, mixing experiments showed that ADAMTS13 activity from NHP was no longer inhibited by addition of heat inactivated remission phase plasma from the patient ( Fig. 1E , R1 and R2). We hypothesized that genetic abnormalities could account for this discrepancy between ADAMTS13 activity and antigen. To investigate this, all 29 exons and exon–intron boundaries of the *ADAMTS13* gene were sequenced. We identified one SNP and one novel mutation on the same allele. The SNP c.2699C > T was located in the T5 domain, which resulted in a substitution from an alanine to a valine at position 900 (p.A900V, Fig. 1F ). The novel mutation c.3530G > A, located in the third linker region of ADAMTS13 between the T8 and CUB1 domain, 25 changed an arginine into a glutamine at position 1177 (p.R1177Q, Fig. 1F ).

### The Novel Mutation p.R1177Q Reduces ADAMTS13 Activity and Has No Effect on Secretion and Conformation

Since mutations as well as SNPs can influence ADAMTS13 secretion and/or activity, 6 the presence of p.A900V and p.R1177Q might explain the subnormal ADAMTS13 activity observed in the patient plasma during remission ( Fig. 1A ). To investigate this, we expressed the p.A900V and p.R1177Q ADAMTS13 variants and studied their effect on ADAMTS13 secretion and activity.

**Fig. 2:**
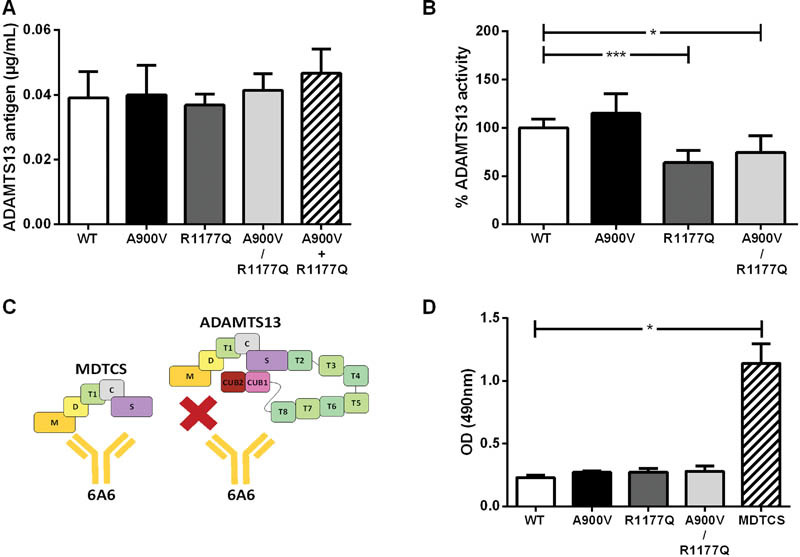
No effect on ADAMTS13 secretion and conformation, but decreased ADAMS13 activity by p.R1177Q mutation. ( **A** ) CHO cells were transiently transfected with plasmids pcDNA6.1-ADAMTS13-WT, pcDNA6.1-ADAMTS13-p.A900V, pcDNA6.1-ADAMTS13-p.R1177Q, pcDNA6.1-ADAMTS13-p.A900V/p.R1177Q or pcDNA6.1-ADAMTS13-p.A900V + pcDNA6.1-ADAMTS13-p.R1177Q, and a pmaxGFP plasmid to determine transfection efficiency. ADAMTS13 antigen levels in the expression medium were determined through ELISA. NHP was used as a reference and set as 1 µg/mL. Kruskal–Wallis compared with WT ( *n* = 3). ( **B** ) A FRETS-VWF73 assay was performed to determine activity of WT and p.A900V, p.R1177Q, and p.A900V/R1177Q ADAMTS13 variants. Kruskal–Wallis compared with WT, * *p* = 0.0283, ** *p* = 0.0005 ( *n* = 9–14). ( **C** ) The cryptic epitope of antibody 6A6 in the metalloprotease domain of ADAMTS13 is accessible in the truncated ADAMTS13 variant MDTCS (ADAMTS13 without T2 till CUB domains; left), but not in full length ADAMTS13 (right). ( **D** ) Conformation of ADAMTS13 variants was studied by adding WT, p.A900V, p.R1177Q, and p.A900V/p.R1177Q ADAMTS13 (6.25nM) to coated 6A6. MDTCS was used as a positive control. Kruskal–Wallis compared with WT, *p* < 0.05 ( *n* = 3).

The p.A900V SNP and p.R1177Q mutations were introduced in the pcDNA6.1-ADAMTS13-WT plasmid, both individually and together by mutagenesis. After transient transfection of CHO cells, we checked whether the substitutions (p.A900V, p.R1177Q, and p.A900V/p.R1177Q) had an influence on ADAMTS13 secretion. Transfection efficiencies were comparable (mean between 19 and 26%, *p* = not significant) for all conditions (data not shown) and expression levels did not differ ( Fig. 2A ), showing that the p.A900V and p.R1177Q substitutions did not induce a secretion defect. Generation of stable cell lines allowed purification of WT, p.A900V, p.R1177Q, and p.A900V/p.R1177Q ADAMTS13 proteins to test their activity using the FRETS-VWF73 assay. The p.A900V SNP had no effect on ADAMTS13 activity. However, ADAMTS13 activity was decreased by 36% due to the single p.R1177Q mutation ( *p* = 0.0005; Fig. 2B ) and by 26% due to the double p.A900V/p.R1177Q mutations ( *p* = 0.0283; Fig. 2B ).

In addition, since the mutation p.R1177Q is located in the third linker region ( Fig. 1F ), this mutation could influence ADAMTS13 conformation. 25 Indeed, we recently showed that changes in the linker regions in ADAMTS13 expose a cryptic epitope in the metalloprotease domain. 25 Therefore, we tested whether the p.A900V SNP or the p.R1177Q mutation could expose the cryptic epitope in the metalloprotease domain, which is shielded by the T2-CUB2 domains in WT ADAMTS13 ( Fig. 2C ). The cryptic epitope in the metalloprotease domain is recognized by antibody 6A6 and is accessible in the MDTCS variant but not in WT ADAMTS13 ( Fig. 2C ). 25 Neither the p.A900V SNP nor the p.R1177Q mutation induced an exposure of the cryptic epitope for 6A6 ( Fig. 2D ). In contrast, 6A6 readily bound to MDTCS with its exposed 6A6 cryptic epitope ( Fig. 2C, D ). In conclusion, the mutation p.R1177Q decreases ADAMTS13 activity and can therefore partially explain subnormal ADAMTS13 activity levels during remission, while ADAMTS13 secretion and conformation are not influenced by the SNP p.A900V and/or mutation p.R1177Q.

## Discussion

We investigated a case of pregnancy-onset TTP using both the plasma of the patient and recombinant proteins to unequivocally determine the diagnosis of immune-mediated or congenital TTP. Correct diagnosis is crucial because therapeutic decisions are based on this. 11 During the acute phase, an ADAMTS13 activity level below 10% was found in the patients' plasma ( Fig. 1A ) and could be ascribed to the presence of inhibitory anti-ADAMTS13 autoantibodies ( Fig. 1E ), resulting in the clear diagnosis of immune-mediated pregnancy-onset TTP.

Surprisingly, however, upon remission, the patient still had subnormal ADAMTS13 activity levels (15–20%) despite normal ADAMTS13 antigen levels (±  60%). This observation could not be explained by the presence of inhibitory anti-ADAMTS13 autoantibodies ( Fig. 1C – 1E ). Genetic analysis revealed that one allele contained a heterozygous SNP c.2699C > T (p.A900V), located in the beginning of the T5 domain and a heterozygous mutation c.3530G > A (p.R1177Q) located in the third linker region of ADAMTS13 ( Fig. 1F ). The p.A900V SNP is a common SNP found in approximately 8% of the normal population and in several TTP patients, 5 6 including cases of pregnancy-onset TTP. 9 11 26 A neutral effect of this SNP was predicted, as this valine is present in C57BL/6J mice. 27 Indeed, our present findings as well as the study of Edwards et al 28 show that the p.A900V SNP does not affect ADAMTS13 secretion or ADAMTS13 activity.

The p.R1177Q mutation, on the other hand, has not been described before and has an allele frequency of < 0.01 according to the Ensembl database ( www.ensembl.org ). This mutation did not affect ADAMTS13 secretion, but had a negative effect on ADAMTS13 activity. The heterozygous nature of the patients' p.A900V/p.R1177Q would, however, predict at least 50% ADAMTS13 activity. However, during remission, ADAMTS13 activity is significantly less than what would be expected from the circulating antigen. Whether undetectable (inhibitory) anti-ADAMTS13 autoantibodies are still present during remission ( Fig. 1C–E ), or whether other factors are involved, remains to be determined. The mutation p.R1177Q is situated in the third linker region between the T8 and CUB1 domain and can make us learn more about the mode of action of ADAMTS13, as we previously showed that the third linker region allows for flexibility in ADAMTS13 which can lead to exposure of a cryptic epitope in the metalloprotease domain of ADAMTS13 ( Fig. 2C ). However, the change of R1177 to Q did not induce such a conformational change in ADAMTS13 ( Fig. 2D ).

Our patient is the fourth reported case of immune-mediated TTP where besides anti-ADAMTS13 antibodies, heterozygous mutations were also identified. In the three patients previously reported by Camilleri et al, the heterozygous R1060W mutation was detected, which affects secretion but not activity, although anti-ADAMTS13 antibodies were the root cause of the TTP episode. 9 Also in our case of pregnancy-onset TTP, the development of anti-ADAMTS13 autoantibodies led to a significant reduction of ADAMTS13 activity allowing precipitation of the disease. Hence, screening for the presence of anti-ADAMTS13 antibodies is, as already known, needed to differentiate between the diagnoses of congenital or immune-mediated TTP. Additional genetic analysis can add value by confirming this diagnosis and could reveal interesting novel substitutions. Those substitutions can give insight into the possible ADAMTS13 deficiencies during remission or can make us learn more about the mode of action of ADAMTS13.

In conclusion, we described a case of pregnancy-onset immune-mediated TTP accompanied with substitutions (p.A900V and p.R1177Q) in ADAMTS13, which do not influence ADAMTS13 secretion or conformation and can only partially explain the subnormal ADAMTS13 activity levels during remission.
